# Interim 2023/24 influenza A vaccine effectiveness: VEBIS European primary care and hospital multicentre studies, September 2023 to January 2024

**DOI:** 10.2807/1560-7917.ES.2024.29.8.2400089

**Published:** 2024-02-22

**Authors:** Marine Maurel, Jennifer Howard, Esther Kissling, Francisco Pozo, Gloria Pérez-Gimeno, Silke Buda, Noémie Sève, Adele McKenna, Adam Meijer, Ana Paula Rodrigues, Iván Martínez-Baz, Ivan Mlinarić, Neus Latorre-Margalef, Gergő Túri, Mihaela Lazăr, Clara Mazagatos, Aitziber Echeverria, Stephen Abela, Marc Bourgeois, Ausenda Machado, Ralf Dürrwald, Goranka Petrović, Beatrix Oroszi, Ligita Jancoriene, Alexandru Marin, Petr Husa, Roisin Duffy, Frederika Dijkstra, Virtudes Gallardo García, Luise Goerlitz, Vincent Enouf, Charlene Bennett, Mariëtte Hooiveld, Raquel Guiomar, Camino Trobajo-Sanmartín, Vesna Višekruna Vučina, Tove Samuelsson Hagey, Ana Sofía Lameiras Azevedo, Jesús Castilla, Gerd Xuereb, Bénédicte Delaere, Verónica Gómez, Kristin Tolksdorf, Sabrina Bacci, Nathalie Nicolay, Marlena Kaczmarek, Angela MC Rose, on behalf of the European IVE group

**Affiliations:** 1 Epiconcept, Paris, France; 2 National Centre for Microbiology, National Influenza Reference Laboratory, WHO-National Influenza Centre, Institute of Health Carlos III, Madrid, Spain; 3 CIBER de Epidemiología y Salud Pública (CIBERESP), Institute of Health Carlos III, Madrid, Spain; 4 National Centre for Epidemiology, Institute of Health Carlos III, Madrid, Spain; 5 Department for Infectious Disease Epidemiology, Respiratory Infections Unit, Robert Koch Institute, Berlin, Germany; 6 Sorbonne Université, INSERM, Institut Pierre Louis d'épidémiologie et de Santé Publique (IPLESP UMRS 1136), Paris, France; 7 HSE Health Protection Surveillance Centre, Dublin, Ireland; 8 National Institute for Public Health and the Environment, Centre for Infectious Diseases Control, Bilthoven, the Netherlands; 9 Epidemiology Department, Instituto Nacional de Saúde Doutor Ricardo Jorge, Lisbon, Portugal; 10 Instituto de Salud Pública de Navarra – IdiSNA – CIBERESP, Pamplona, Spain; 11 Croatian Institute of Public Health, Zagreb, Croatia; 12 Public Health Agency of Sweden, Stockholm, Sweden; 13 National Laboratory for Health Security, Epidemiology and Surveillance Centre, Semmelweis University, Budapest, Hungary; 14 Cantacuzino National Military Medical Institute for Research and Development, Bucharest, Romania; 15 Infectious Disease Prevention and Control Unit (IDCU), Health Promotion and Disease Prevention, Msida, Malta; 16 Department of Infectious Diseases, CHU UCL Namur (site Godinne), Université catholique de Louvain, Yvoir, Belgium; 17 National Reference Centre for Influenza, Robert Koch Institute, Berlin, Germany; 18 Clinic of Infectious Diseases and Dermatovenerology, Institute of Clinical Medicine, Medical Faculty, Vilnius University, Vilnius, Lithuania; 19 Dr Victor Babes Clinical Hospital of Infectious and Tropical Diseases, Bucharest, Romania; 20 University Hospital Brno and Masaryk University, Brno, Czechia; 21 Servicio de Vigilancia y Salud Laboral, Dirección General de Salud Pública y Ordenación Farmacéutica, Consejería de Salud y Consumo, Andalucía, Spain; 22 Centre National de Référence Virus des Infections Respiratoire (CNR VIR), Institut Pasteur Université Paris Cité, Paris, France; 23 National Virus Reference Laboratory, University College Dublin, Dublin, Ireland; 24 Nivel, Utrecht, the Netherlands; 25 Laboratório Nacional Referência Gripe e outros Vírus Respiratórios, Instituto Nacional de Saúde Doutor Ricardo Jorge, Lisbon, Portugal; 26 Servicio de vigilancia y control epidemiológico, Subdirección general de Epidemiología y Vigilancia de la Salud, Dirección General de Salud pública, Valencia, Spain; 27 Department of Child and Adolescent Health, Mater Dei Hospital, Msida, Malta; 28 European Centre for Disease Prevention and Control, Stockholm, Sweden; 29 The members of the European IVE group are listed under Acknowledgements.

**Keywords:** influenza, vaccine effectiveness, multicentre study, test-negative design, Europe

## Abstract

Influenza A viruses circulated in Europe from September 2023 to January 2024, with influenza A(H1N1)pdm09 predominance. We provide interim 2023/24 influenza vaccine effectiveness (IVE) estimates from two European studies, covering 10 countries across primary care (EU-PC) and hospital (EU-H) settings. Interim IVE was higher against A(H1N1)pdm09 than A(H3N2): EU-PC influenza A(H1N1)pdm09 IVE was 53% (95% CI: 41 to 63) and 30% (95% CI: −3 to 54) against influenza A(H3N2). For EU-H, these were 44% (95% CI: 30 to 55) and 14% (95% CI: −32 to 43), respectively.

As at week 5 2024, influenza virus is circulating in Europe, with median influenza test positivity among sentinel primary care networks at 34%, and influenza A as the main virus type [1]. Most countries are reporting dominance of influenza A(H1N1)pdm09 virus [1]. Here, we present interim influenza vaccine effectiveness (IVE) from European primary care and hospital multi-country studies between September 2023 and January 2024.

## Influenza vaccination in Europe

In the northern hemisphere for the 2023/24 season, the A/Victoria/4897/2022 (H1N1)pdm09-like clade 5a.2a.1 virus was recommended by the World Health Organization (WHO) as the influenza A(H1N1)pdm09 egg-based vaccine strain virus. For influenza A(H3N2) virus egg-based vaccines, the recommendation was an A/Darwin/9/2021 (H3N2)-like clade 2a.2 virus [2], the same as for 2022/23.

Influenza vaccination target groups in the European Union (EU) include older adults, certain occupational groups, individuals with chronic conditions, and those at increased risk of influenza complications and severe disease [3]. In around one third of European Union/European Economic Area (EU/EEA) countries, vaccination of healthy children is also recommended, e.g. in Ireland among those 2–17 years, Spain among those ≥ 6 months–4 years, and Romania among those ≥ 6 months–17 years.

The I-MOVE (Influenza – Monitoring Vaccine Effectiveness in Europe) primary care (EU-PC) and hospital (EU-H) multi-country studies have estimated IVE since 2008/09 (EU-PC) and 2012/13 (EU-H). These studies are now coordinated through the ECDC Vaccine Effectiveness, Burden and Impact Studies (VEBIS) project. Within these two multi-country studies, we assessed interim IVE for the 2023/24 season. Our results aim to inform the end-February 2024 WHO Vaccine Strain Selection Committee.

## Study design and setting

**Figure 1:**
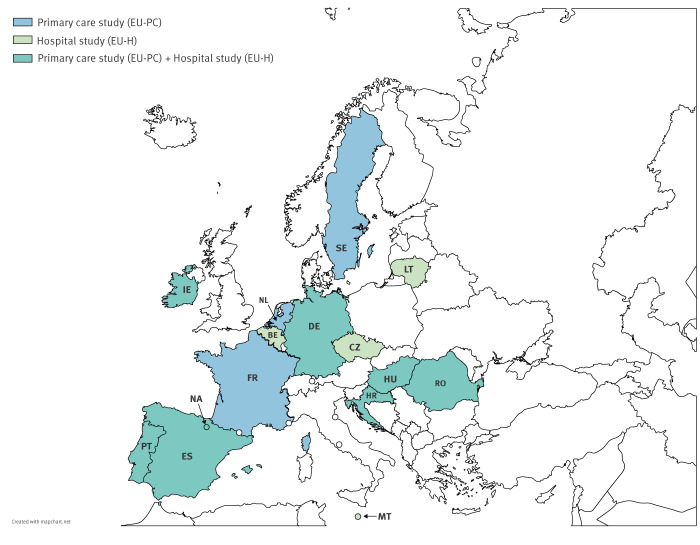
Sites^a^ participating in VEBIS European primary care and hospital influenza vaccine effectiveness studies, September 2023−January 2024 (n  =  15)

BE: Belgium; CZ: Czechia; DE: Germany; ES: Spain (several regions); FR: France; HR: Croatia; HU: Hungary; IE: Ireland; LT: Lithuania; MT: Malta; NA: Navarre (Spain, one region); NL: the Netherlands; PT: Portugal; RO: Romania; SE: Sweden; VEBIS: Vaccine Effectiveness, Burden and Impact Studies.

^a^ Nine sites included in 2023/24 primary care (EU-PC) analysis (providing ≥ 10 influenza cases): DE, ES, FR, HR, IE, NA, NL, PT, SE; seven sites included in 2023/24 hospital (EU-H) analysis (providing ≥ 10 influenza cases): BE, DE, ES, HR, MT, NA, PT.

Nine of 11 EU-PC and seven of 12 EU-H participating study sites in 10 countries reported sufficient influenza cases (≥ 10 eligible influenza cases) to be included in interim IVE analyses (Figure 1).

Using the test-negative design, patients meeting a common case definition were systematically selected for recruitment and swabbing [4,5]. For EU-PC, we included patients meeting the EU acute respiratory infection (ARI) case definition: patients with sudden onset of symptoms and at least one of four respiratory symptoms (cough, sore throat, shortness of breath, coryza). For EU-H, we included patients meeting a severe ARI (SARI) case definition: hospitalised for at least 24 h with at least one of three symptoms (fever, cough, shortness of breath) and with symptom onset within 10 days of hospital admission. Reverse transcription (RT)-PCR testing was used for influenza virus detection and type A subtyping. Patients testing negative were designated as controls, and those testing positive as cases. Controls presenting before the week of onset of the first influenza (sub)type/clade-specific case were excluded from the analysis of that (sub)type. Eight of nine sites in EU-PC and three of seven sites in EU-H selected all (or a random sample of) influenza virus-positive specimens above specified quantification cycle (Cq) values for haemagglutinin genome segment or whole genome sequencing, followed by phylogenetic analysis to determine clade distribution.

## Virological results

**Figure 2:**
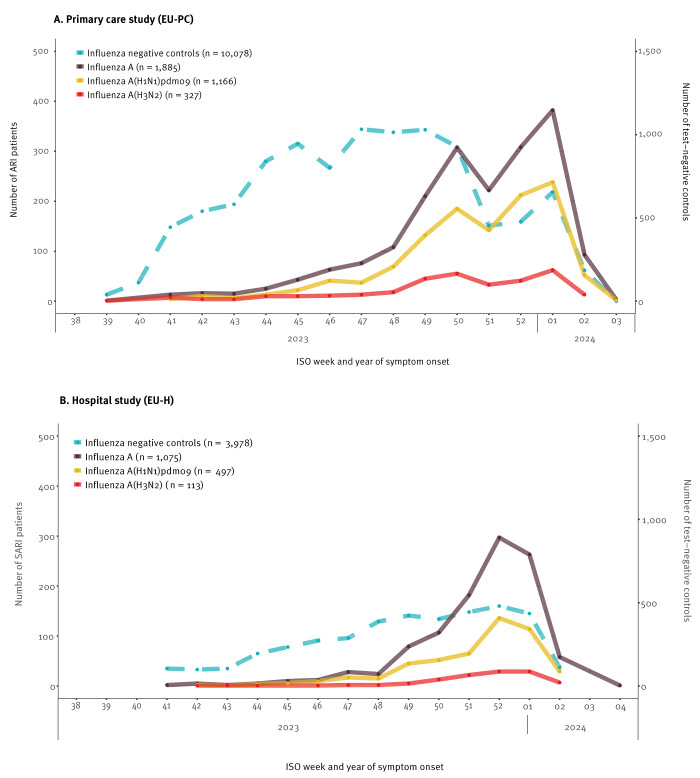
Number of influenza cases and test-negative controls by week of onset, VEBIS European (A) primary care and (B) hospital studies, September 2023–January 2024 (n = 17,016^a^)

ARI: acute respiratory infection; ISO: International Organisation for Standardisation; SARI: severe acute respiratory infection; VEBIS: Vaccine Effectiveness, Burden and Impact Studies.

^a^ Numbers for subtypes A(H1N1)pdm09 and A(H3N2) are subsets of the total number of influenza A cases.

We included 12,036 eligible patients between week 39 2023 and week 3 2024 in the EU-PC study (Figure 2A), and excluded 46 influenza B and 27 influenza virus-positive infections (type unreported). There were 1,885 influenza A virus infections; 368 (20%) were not subtyped. Among 1,517 subtyped viruses, 1,166 (77%) were A(H1N1)pdm09 and 351 (23%) A(H3N2) (we excluded 24 patients from four sites for A(H3N2) analyses due to small sample size).

We included 5,115 eligible SARI patients between week 41 2023 and week 4 2024 in the EU-H study (Figure 2B), and excluded one influenza B and 61 influenza virus-positive (type unreported) infections. There were 1,075 influenza A infections; 500 (47%) were A(H1N1)pdm09, 122 (11%) A(H3N2) and 453 (42%) A not subtyped. Two study sites were excluded from A(H1N1)pdm09 and five from A(H3N2) analyses due to small sample size (three and nine patients, respectively).

Among the 1,166 A(H1N1)pdm09 viruses in EU-PC, 160 (14%) were sequenced. Of these, 56% (90/160) belonged to clade 5a.2a and 44% (70/160) to vaccine clade 5a.2a.1 (Table 1). In EU-H, 20 of 497 (4%) A(H1N1)pdm09 viruses were sequenced; 16 belonged to clade 5a.2a.1, and four to 5a.2a.

**Table 1:** Influenza viruses characterised by genetic clade and amino acid substitutions, VEBIS European primary care and hospital studies, September 2023–January 2024 (n = 222)

Influenza virus	Clade	EU-PC^a^		EU-H^a^	
		n	%	n	%
Influenza A(H1N1)pdm09					
Total number		1,166		497	
Sequenced		160	14	20	4
AH1/Sydney/5/2021-like	5a.2a	90	56	4	NC
AH1/Victoria/4897/2022-like	5a.2a.1	70	44	16	NC
+ R113K	5a.2a.1 + R113K	28	40	12	NC
+ R45K	5a.2a.1 + R45K	29	41	4	NC
Influenza A(H3N2)					
Total number		327		113	
Sequenced		41	13	1	1
AH3/Thailand/8/2022-like	2a.3a.1	41	NC	1	NC
+ N122D	2a.3a.1 + N122D	24	NC	0	NC
+ N122D + K276E	2a.3a.1 + N122D + K276E	21	NC	0	NC

EU-PC: European primary care multicentre VEBIS study; EU-H: European hospital multicentre VEBIS study; NC: not calculated (percentages not shown where denominators < 60); VEBIS: Vaccine Effectiveness, Burden and Impact Studies.

^a^ At time of publishing (Feb 2024), not all specimens from the study period were processed.

All 41 (13%) A(H3N2) viruses sequenced in EU-PC and the one in EU-H belonged to the 2a.3a.1 clade (Table 1). Among sequenced EU-PC A(H3N2) viruses, 24 had the N122D substitution.

## Vaccination definitions and patient characteristics

Vaccination information was obtained through GP or hospital records, by data linkage with national influenza vaccination databases, or patient interview, depending on the study site. We defined vaccinated patients as those having received 2023/24 influenza vaccine at least 14 days before onset of symptoms, and excluded those vaccinated < 14 days before symptom onset.

In EU-PC, 13% (1,307/10,078) of influenza virus A test-negative controls were vaccinated, vs 10% (119/1,166) and 11% (37/327) of influenza A(H1N1)pdm09 and A(H3N2) cases, respectively. Among controls, 33% (3,313/10,078) were < 17 years, vs 29% (337/1,166) and 26% (86/327) among influenza A(H1N1)pdm09 and A(H3N2) cases, respectively. In EU-H, 40% (1,595/3,978) of controls and 39% (193/497) influenza A(H1N1)pdm09 cases were vaccinated vs 57% (64/113) of A(H3N2) cases. Sixty-four percent (2,544/3,978) of controls in the EU-H study were ≥ 65 years, vs 62% (309/497) and 75% (85/113) of influenza A(H1N1)pdm09 and A(H3N2) cases, respectively (Table 2).

**Table 2:** Interim vaccine effectiveness against influenza A, A(H1N1)pdm09 and A(H3N2), by age group and target group for vaccination, VEBIS European primary care and hospital studies, September 2023–January 2024

Influenza (sub)type/clade and study	Study population^a^	Cases			Controls			IVE^c^	95% CI
		All	Vacc	%	All	Vacc^b^	%		
**Influenza A**									
EU-PC	All ages	1,885	192	10	10,078	1,307	13	51	41 to 59
	0–17 years	516	24	5	3,313	279	8	71	55 to 82
	18–64 years	1,192	82	7	5,310	391	7	40	22 to 55
	≥ 65 years	177	86	49	1,455	640	44	45	22 to 62
	Target group^d^	761	151	20	4,458	1,122	25	53	42 to 63
EU-H	All ages	1,075	450	42	3,978	1,595	40	38	27 to 48
	18–64 years	281	51	18	708	152	21	53	31 to 68
	≥ 65 years	703	392	56	2,544	1,363	54	36	22 to 47
	Target group^d^	978	441	45	3,600	1,566	44	40	28 to 49
**Influenza A(H1N1)pdm09**									
EU-PC	All ages	1,166	119	10	9,835	1,301	13	53	41 to 63
	0–17 years	337	9	3	3,234	276	9	85	71 to 93
	18–64 years	716	53	7	5,165	390	8	40	17 to 57
	≥ 65 years	113	57	50	1,436	635	44	41	8 to 62
	Target group^d^	460	93	20	4,382	1,116	25	54	40 to 65
EU-H	All ages	497	193	39	3,670	1,550	42	44	30 to 55
	18–64 years	138	19	14	661	150	23	59	30 to 77
	≥ 65 years	309	171	55	2,356	1,320	56	41	23 to 54
	Target group^d^	442	188	43	3,345	1,521	45	47	33 to 58
**Influenza A(H1N1)pmd09 clade 5a.2a**									
EU-PC	All ages	90	8	13	7,919	1,100	14	52	−7 to 78
**Influenza A(H1N1)pmd09 clade 5a.2a.1**									
EU-PC	All ages	70	7	10	7,392	1,081	15	39	−44 to 74
**Influenza A(H3N2)**									
EU-PC	All ages	327	37	11	9,442	1,196	13	30	−3 to 54
	18–64 years	211	15	7	4,877	351	7	35	−13 to 65
	Target group^d^	145	31	21	4,187	1,034	25	30	−10 to 57
EU-H	All ages	113	64	57	3,186	1,436	45	14	−32 to 43
	≥ 65 years	85	57	67	2,128	1,236	58	13	−42 to 45
	Target group^d^	108	64	59	2,969	1,417	48	13	−34 to 43

CI: confidence interval; EU-H: European hospital multi-country VEBIS study; EU-PC: European primary care multi-country VEBIS study; IVE: influenza vaccine effectiveness; Vacc: vaccinated; VEBIS: Vaccine Effectiveness, Burden and Impact Studies.

Sites included in EU-PC analysis against influenza A and A(H1N1)pdm09: Croatia, France, Germany, Ireland, the Netherlands, Portugal, Navarre, Spain and Sweden. Against influenza A(H3N2): France, Germany, Ireland, the Netherlands and Spain.

Sites included in EU-H analysis of IVE against influenza A and influenza A(H1N1)pdm09: Belgium, Croatia, Germany, Malta, Navarre, Portugal and Spain. Against influenza A(H3N2): Navarre and Spain.

^a^ Age-specific or target group-specific IVE was not included for overall or (sub)type-specific IVE in some study sites, where sample size did not allow estimation of IVE.

^b^ Vaccine brand information was available for 77% (1,005/1,307) of vaccinated controls in EU-PC: all vaccines were quadrivalent, 9% (n = 88) were cell-based, 1% (n = 14) were live attenuated influenza vaccine (LAIV), 5% (n = 53) were adjuvanted vaccine, 12% (n = 118) were high-dose vaccine, 73% (n = 732) were unadjuvanted, standard dose, egg-propagated, inactivated. In EU-H 77% (1,230/1,595) of vaccinated controls had available vaccine brand information: all vaccines were quadrivalent, 61% (n = 749) egg-propagated, inactivated, unadjuvanted, standard dose vaccines, 18% (n = 221) were high dose, 12% (n = 145) adjuvanted, 8% (n = 101) cell-based and 1% (n = 14) LAIV.

^c^ The IVE models were adjusted by study site, age, sex, presence of chronic conditions and onset date. The best functional forms of the continuous variables age and onset date (categories, splines, linear terms) were selected using the Akaike information criterion.

^d^ Groups targeted by seasonal influenza vaccination include older adults (aged 55, 60 or 65 years or older), those in medical risk groups and healthy children (in Ireland among those aged 2–17 years and in Spain among those aged ≥ 6 months to 4 years), as defined locally in the studies and study sites.

## Influenza vaccine effectiveness

Using logistic regression, we estimated an odds ratio (OR) for vaccination adjusted for a priori potential confounding variables of study site, age, sex, presence of chronic conditions and onset date. We calculated VE as 1-OR x 100. For models with small sample sizes (< 10 cases or controls per parameter), we performed a sensitivity analysis using Firth’s penalised logistic regression [6]. Where differences between this and the original estimate were ≥ 10%, estimates were not presented.

For all ages, IVE against influenza A was 51% (95% confidence interval (CI): 41 to 59) in EU-PC and 38% (95% CI: 27 to 48) in EU-H (Table 2).

In EU-PC, all-age VE against influenza A(H1N1)pdm09 was 53% (95% CI: 41 to 63). Among children, IVE was 85% (95% CI: 71 to 93). All-age IVE was 52% (95% CI: −7 to 78) and 39% (95% CI: −44 to 74) against clade 5a.2a and 5a.2a.1, respectively. In EU-H, all-age influenza A(H1N1)pdm09 VE was 44% (95% CI: 30 to 55).

The all-age VE against influenza A(H3N2) in EU-PC was 30% (95% CI: −3 to 54) and 14% (95% CI: −32 to 43) in EU-H.

## Discussion

Our results from two well-established European multi-country studies indicate 2023/24 interim VE estimates against influenza A in primary care and hospital settings were 51% and 38% among all ages, and 53% and 40% among vaccination target groups, respectively. Point estimates against influenza A(H1N1)pdm09 ranged between 40 and 54% among adults in EU-PC; between 41 and 59% in EU-H. In EU-PC, A(H1N1)pdm09 IVE among children was high (85%).

Influenza virus A(H1N1)pdm09 5a.2a.1 and 5a.2a clades both circulated in the 2022/23 and 2023/24 seasons in Europe [7,8], with WHO vaccine clade recommendations changing from 5a.2a for the 2022/23 season to 5a.2a.1 for the 2023/24 season [2,9]. The change in vaccine clade may have contributed to higher IVE estimates in 2023/24 over 2022/23. Point estimates indicated a higher IVE against clade 5a.2a than 5a.2a.1, but confidence intervals overlapped. A higher proportion of children had 5a.2a infections (36% vs 26%), although sample size was too small for age-stratified clade-specific analyses, which might help disentangle the age-vs-clade effect. Interim 2023/24 IVE results in Canada report comparable differences in point estimates between clades [10]. Despite genetic diversity within circulating 2023/24 A(H1N1)pdm09 viruses, antigenic studies demonstrate good recognition of 5a.2a and 5a.2a.1 test viruses by the vaccine strain [7].

The 2023/24 interim IVE point estimates against influenza A(H3N2) were 30–35% in EU-PC; similar to the end-of-season estimate against A(H3N2) in 2022/23 [11]. For EU-H, the interim 2023/24 point estimates of 13–14% against influenza A(H3N2) were lower than the estimates of 20–25% in the 2022/23 season [12]. Vaccine clades were the same in both seasons (2a.2), but circulating clades differed, with 2b predominating in 2022/23 and 2a.3a.1 in 2023/24 [7,8]. More than half of sequenced 2a.3a.1 viruses harboured the N122D substitution, associated with a potential loss of a glycosylation site, which may affect IVE. Antigenic studies showed variable recognition of the 2023/24 circulating viruses [7]. Studies of early or interim IVE estimates from Canada reported comparable, but slightly higher IVE against both influenza virus subtypes [10,13].

Limitations include small sample size for IVE against A(H3N2), as influenza A(H1N1)pdm09 circulation has dominated the season thus far, resulting in low precision and some stratified estimates unable to be calculated. The low proportion of available sequenced viruses reduced precision for clade-specific IVE estimates for A(H1N1)pdm09. As with all observational studies, unmeasured confounding and selection bias cannot be ruled out. The influenza season continues in Europe, and end-of-season estimates will provide more precise IVE estimates against circulating influenza strains and clades.

## Conclusion

Overall, up to 53% and 44% of vaccinated individuals in primary care or hospital settings, respectively, were protected against mild and severe influenza during the 2023/24 season. Influenza vaccination should be promoted in line with national guidelines and recommendations in all European countries with ongoing influenza virus circulation.
